# Quality Assessment for PCR-based Minimal Residual Disease in Lymphoma: 10 Years of Cross-laboratory Standardization Process Within the Fondazione Italiana Linfomi MRD Network

**DOI:** 10.1097/HS9.0000000000000639

**Published:** 2021-09-06

**Authors:** Barbara Mantoan, Elisa Genuardi, Martina Ferrante, Irene Della Starza, Elena Ciabatti, Susanna Grassi, Lucia Anna De Novi, Marzia Cavalli, Claudia Mannu, Anna Gazzola, Riccardo Bomben, Massimo Degan, Beatrice Alessandria, Christiane Pott, Marie-Hélène Delfau-Larue, Ramon García-Sanz, Claudio Agostinelli, Valter Gattei, Sara Galimberti, Ilaria Del Giudice, Gianluca Gaidano, Marco Ladetto, Simone Ferrero, Daniela Drandi

**Affiliations:** 1 Department of Molecular Biotechnologies and health sciences, Hematology Division, University of Torino, Italy; 2 Hematology, Department of Translational and Precision Medicine, Sapienza University of Roma, Italy; 3 Gimema Foundation, Rome, Italy; 4 Division of Hematology, Department of Clinical and Experimental Medicine, Santa Chiara Hospital, Pisa, Italy; 5 Hematopathology section, IRCCS, Azienda Ospedaliero-Universitaria di Bologna, University of Bologna, Italy; 6 Clinical and Experimental Onco-Hematology Unit, Centro di Riferimento Oncologico, I.R.C.C.S., Aviano, Italy; 7 Second Medical Department, University Hospital Schleswig-Holstein, Kiel, Germany; 8 Laboratoire d’immunologie biologique, hôpital Henri-Mondor, Créteil Cedex, France; 9 Division of Hematology, Servicio de Hematología, Hospital Universitario de Salamanca, Spain; 10 Division of Hematology, Department of Translational Medicine, Università del Piemonte Orientale, Novara, Italy; 11 SC Ematologia and Infrastruttura Ricerca Formazione Innovazione, Az Ospedaliera SS Antonio e Biagio e Cesare Arrigo, Alessandria, Italy; 12 Hematology U, “AOU Città della Salute e della Scienza di Torino,” Italy

Three decades ago, the first PCR-based approach for monoclonal detection in B-lymphoproliferative disorders was published.^1^ Nowadays, a real-time quantitative polymerase chain reaction (qPCR) is the gold standard for minimal residual disease (MRD) monitoring in non-Hodgkin lymphoma (NHL),^2^ and it is a clinically useful tool for early identification of drug response, long-term outcome prediction, and patients stratification in highly effective treatments programs within clinical trials.^3^

PCR-based MRD analysis requires extensive knowledge in laboratory processes and data interpretation. Usage of standardized assays and internationally accepted guidelines for data analysis are mandatory, especially in the contest of multicenter clinical trials. Standardization of procedures should be the main aim of translational research laboratories, focused on MRD analysis, and quality assessments are essential to estimate the laboratory performance to improve efficiency and to define robust criteria capable to guarantee common results worldwide.

This behavior is exemplified by the activities conducted, over the past years, by the European consortium for acute lymphoblastic leukemias and lymphomas and by the Fondazione Italiana Linfomi (FIL MRD network) study group (Supplemental Digital Figure 1S, http://links.lww.com/HS/A193).

We here report an overview of a 10-year standardization program performed by the Italian FIL MRD Network, a group of 4 Italian hematology laboratories collaborating for the MRD monitoring in patients with lymphoma. This letter reports the challenges achieved by the MRD Network, with the intention to provide a practical guide for all laboratories motivated to setup standardized procedures and streamline quality control (QC) assessments for marker screening and MRD analysis and that might join the MRD network, in the future.

Since 2009, the 4 Italian laboratories which constitute the MRD Network (Turin, Rome, Pisa, and Bologna, the latter replaced by Aviano since 2017) established standardized workflows for PCR-based MRD monitoring of patients affected by follicular lymphoma (FL) or mantle cell lymphoma (MCL). Therefore, since 2012, the MRD Network has been performing MRD analysis in the contest of prospective lymphoma clinical trials, sponsored by FIL (www.filinf.it) (Supplemental Digital Table 1S, http://links.lww.com/HS/A193), for whom blood samples are centralized to the 4 laboratories, based on the geographical distribution of the Italian hematology centers (Supplemental Digital Figure 1S, http://links.lww.com/HS/A193).

The main objectives of the MRD Network are (1) the harmonization and standardization of procedures and PCR, according to the EuroMRD guidelines^4^; (2) the knowledge sharing of MRD analysis especially with laboratories yet involved in EuroMRD group; and (3) the development of new highly sensitive methods for marker screening and MRD monitoring.

To achieve and constantly verify the reproducibility of results among the 4 laboratories the Network routinely organizes QC assessments involving distinct tasks (Supplemental Digital Table 2S, http://links.lww.com/HS/A193). The tasks are performed independently by each laboratory, according to previously described PCR protocols and following the EuroMRD guideline.^1,2,4–7^ Each laboratory takes turn in QC organization that consists in task setup, samples shipment, and data analysis followed by discussion during the biyearly meetings, in which all laboratories results are compared with the “reference” results provided by the QC organizer.

In the last 10 years, 16 QC rounds have been performed including the starting QC rounds focused on the harmonization of DNA extraction and setup of PCR methods.

Altogether, 208 samples have been analyzed. gDNA from 126 FL and 82 MCL patients were analyzed for marker detection (*IGH-VDJ* rearrangements, *BCL1/IGH* or *BCL2/IGH* fusion genes) by nested-PCR and/or qPCR. Since 2015, an additional task, based on droplet digital PCR (ddPCR), was introduced and QCs were performed on 52 samples (42 FL and 10 MCL). Tasks performance was evaluated in terms of laboratories concordance to the “reference” value, based on predefined scores (Supplemental Digital Table 3S, http://links.lww.com/HS/A193).

Here, each single task is described and discussed.

Task 1 consisted in raw data analysis (5–10 qPCR reactions) carried out in accordance with the EuroMRD guidelines.^4^ Only 2 of 16 QCs (QC1 and QC11), scored as reported in Supplemental Digital Table 3S, http://links.lww.com/HS/A193, showed >10% discordant interpretations (Supplemental Digital Table 4S, http://links.lww.com/HS/A193). Overall, a good concordance rate was observed, and it was maintained during the following QC rounds.

Task 2 was focused on the detection of *BCL2/IGH* rearrangement, for the major breakpoint region (*MBR*), by nested-PCR.^1^ Only 1/16 QCs showed more than 10% of discordant interpretations (QC3, Supplemental Digital Table 4S, http://links.lww.com/HS/A193). One hundred twenty samples (472 PCR reactions) were analyzed: 59 MRD positive, 37 MRD negative, and 24 straddling the detection limit (SDL). Seven of 120 samples showed discordances between laboratories (totally 9/472 PCR reactions) not linked to only one laboratory. Among the 24 SDL samples, 11 were concordantly positive (7) or negative (4) and 13 still SDL (showing alternatively positive and negative results) and 10/13 shown concordant PCR in at least two laboratories.

Task 3 consisted in *BCL2/IGH* MRD qPCR analysis on 111 out of 120 samples analyzed in Task 2: 55 were MRD positive, 32 MRD negative and 24 straddling the quantification limit (SQL). Of note, 20 of 55 MRD positive samples showed discordances in tumor burden quantity, among the 4 laboratories. Discordances in terms of positivity versus negativity were defined as “major,” while were defined “minor” when the positive result remained positive or positive but not quantifiable (PNQ).^4,7^ Based on this classification, only 3 cases were defined as major discordances while 18 as minor: 8 positive samples showed >1 log difference respect to the reference and 10 were PNQ, based on EuroMRD definition.^4^ Of note, one sample showed 1 minor and 1 major PCR discordance (FU2/2) (Figure 1A). Discordances were not laboratory related. Finally, within the 24 SQL samples, 15 were concordantly positive (14) or negative (1) and 5 of 9 showed concordant PCR in 50% of laboratories, while 4 of 9 were discordant in only one laboratory (2 PNQ and 2 negative).

**Figure 1.**
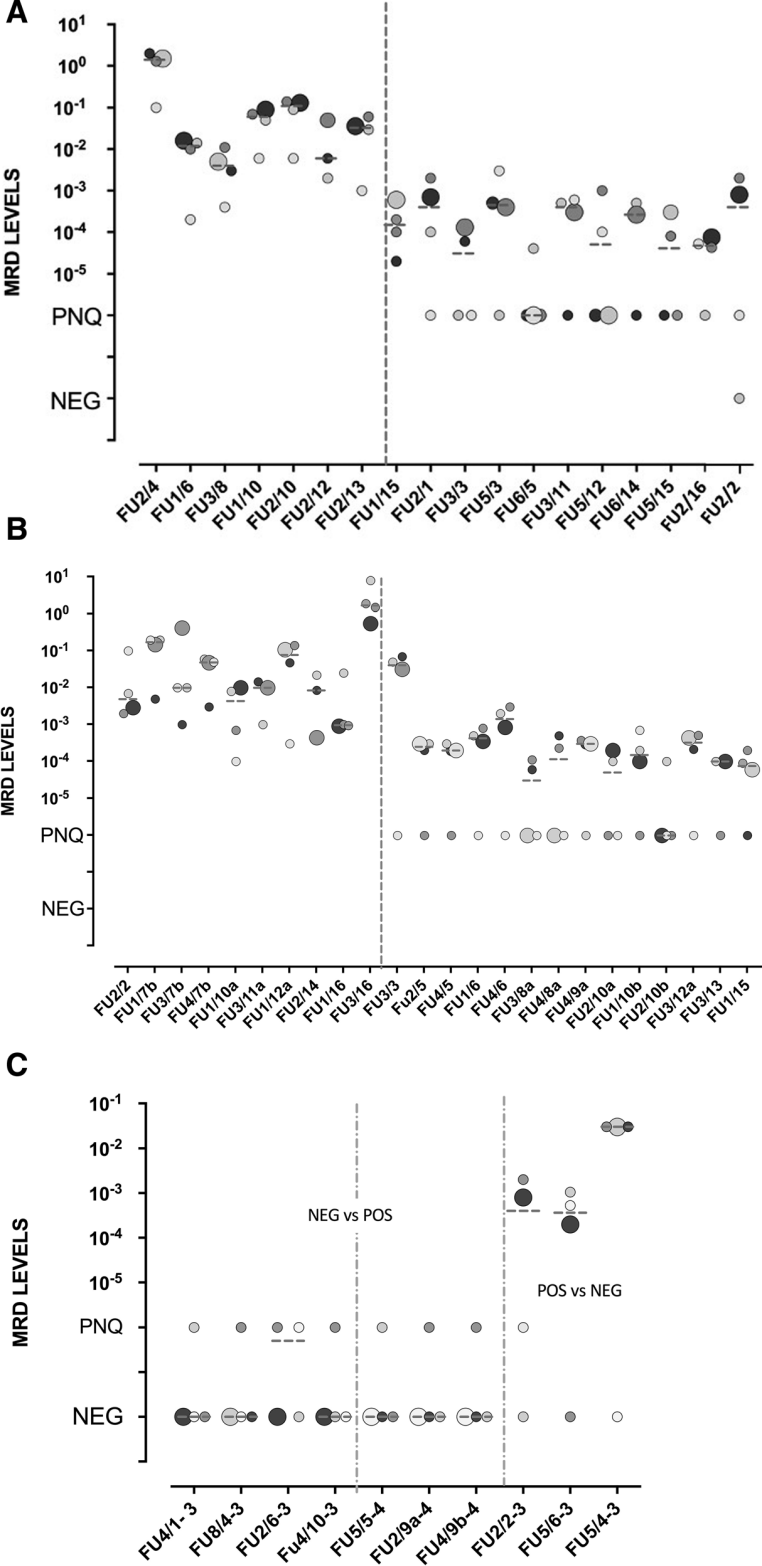
**Discordances in qPCR results observed in 16 QC rounds.** (A) Task 3: Each laboratory is represented by a grayscale-colored dot; bigger dots represent “reference” PCR result; dashed line represents median value; FU samples: FUn/QC (ie, FU2/4 means FU sample number 2 quantified at the QC4). FU2/2 presents also a major discordance see Figure 1C. (B) Task 4. Each laboratory is represented by a dot; bigger dots represent “reference” PCR result; dashed line represents median value; FU samples: FUn/QC. (C) Ten of 208 (4%) major discordances in qPCR results observed in Task 3 and Task 4. On the left and central panel, NEG vs POS: reference negative while discordant PCR are PNQ; on the right panel, discordances POS vs NEG: reference positive and discordant PCR are negative. Each laboratory is represented by a grayscale-colored dot; bigger dots represent “reference” PCR result; FU samples: FUn/QC-Task; NEG vs POS: reference negative while discordant PCR are PNQ; POS vs NEG: reference positive and discordant PCR are negative. FU = follow-up; PCR = polymerase chain reaction; PNQ = positive but not quantifiable.

Task 4 was subdivided in 3 subtasks. Task 4a was about *IGH* sequence analysis and consisted in: (1) sequence analysis and interpretation; (2) sequence description based on EuroMRD guidelines; (3) design of primers and probes for setting an allele patient-specific qPCR (ASO-PCR). Four QC rounds were needed to harmonize all laboratories results (Supplemental Digital Table 4S, http://links.lww.com/HS/A193). Task 4b consisted in MRD analysis performed by nested-PCR on 82 samples (268 PCR reactions): 58 MRD positive, 20 MRD negative, and 4 SQL. All SQL samples showed PCR results concordant in 50% of laboratories, underlining the unreproducible nature of these type of samples due to their low tumor infiltration.^8^ Overall, 12 of 15 QC rounds were fully concordant (Supplemental Digital Table 4S, http://links.lww.com/HS/A193). Only 3 QCs showed discordances in 5 of 24 samples (9/88 PCR reactions), 6 false positive, and 3 false-negative, mostly in QC 9. Task 4c consisted in qPCR analysis performed on the same 82 FU from TASK4b. Minor discordances were observed in 24 of 58 MRD positive samples: 10 of them were positive with >1 log of differences compared to the reference value, and 14 were PNQ (Figure 1B).

Among the MRD negative samples, 3 major discordances were observed (cases 5, 6, 7; Figure 1C). Again, any discordance was laboratory-related.

In 2015, 2 new tasks, based on the ddPCR approach, were introduced (Tasks 5 and 6) and in 2016, 3 international EuroMRD groups (Kiel (DE), Créteil (F), Salamanca (ES)), joined the MRD network contributing to its harmonization activity. The ddPCR tasks (5 and 6) were performed on the same samples already tested in Tasks 2 and 3 and showed a good rate of concordance (Supplemental Digital Table 4S, http://links.lww.com/HS/A193) among laboratories. Along all QCs, ddPCR identified false positive qPCR results (PNQ by qPCR but negative by nested-PCR) and was able to quantify a good rate of PNQ samples (Figure 2).

**Figure 2.**
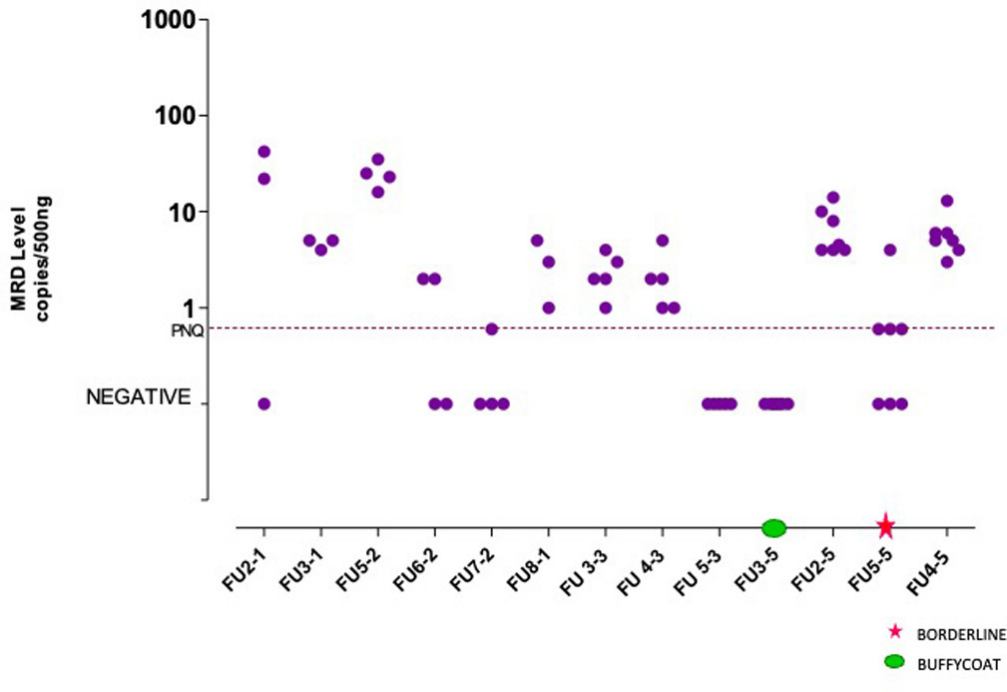
**Task 5 analysis.** qPCR/PNQ samples evaluated by ddPCR along 5 QC rounds. dd = droplet digital; PCR = polymerase chain reaction; PNQ = positive but not quantifiable. q = quantitative.

This experience allowed the MRD Network to consolidate and verify the quality and competences of all laboratories involved in MRD analysis allowing a significant improvement of the performance over the years.^8–10^ As reported in Supplemental Digital Table 4S, http://links.lww.com/HS/A193, the concordance of QC rounds among laboratories has progressed along the years.

A list of the publications generated in the context of the activities of the FIL MRD network is provided (Supplemental Digital Table S5, http://links.lww.com/HS/A193).

qPCR and ddPCR tasks were the most challenging and thanks to this pivotal experience all the laboratories developed a high level of reproducibility and are now skilled for standardization processes and actively involved in work-packages for new technologies (ie, dPCR and NGS) within the EuroMRD consortium.

Of note, thanks to the activity of the Network, the first results of FIL MRD-driven clinical trials have been recently presented and new collaborative studies have been published.^11–13^

Finally, the recent pandemic events pointed out the usefulness of having a network with interchangeable laboratories that guarantee the continuous analysis of patient samples. A contingency plan has been established in 2020, allowing an alternative dislocation of samples among the 4 laboratories in case of need.

In conclusion, the implementation of molecular tests for clinical use involves many levels of assessment. The interlaboratories reproducibility is a mandatory requisite that must be tracked, especially among laboratories involved in disease monitoring in the context of multicenter studies. We showed that a network of laboratories sharing standardized procedures and analysis is mandatory and feasible in the context of a multicenter scientific group, as FIL. The further challenges of network will be to provide a practical guide for those laboratories willing to set QC assessments for marker screening and MRD analysis, also outside the FIL clinical trials, or that might be interested to join our network for improving the reliability of molecular MRD monitoring in lymphomas.

## Disclosures

The authors have no conflicts of interest to disclose.

## Sources of funding

This study was supported by Fondi di Ricerca Locale, Università degli Studi di Torino, Italy, and Fondazione CRT (projects code: 2018.1284), Turin, Italy.

## Supplementary Material


