# Supplementary material for: Quality Assessment for PCR-based Minimal Residual Disease in Lymphoma: 10 Years of Cross-laboratory Standardization Process Within the Fondazione Italiana Linfomi MRD Network
Source: Hemasphere. 2021 Sep 6;5(10):e639. doi: 10.1097/HS9.0000000000000639 (PMC8423385; doi:10.1097/HS9.0000000000000639)
Supplement: Supplementary file 1 [file hs9-5-e639-s001.pptx]

## Slide 1
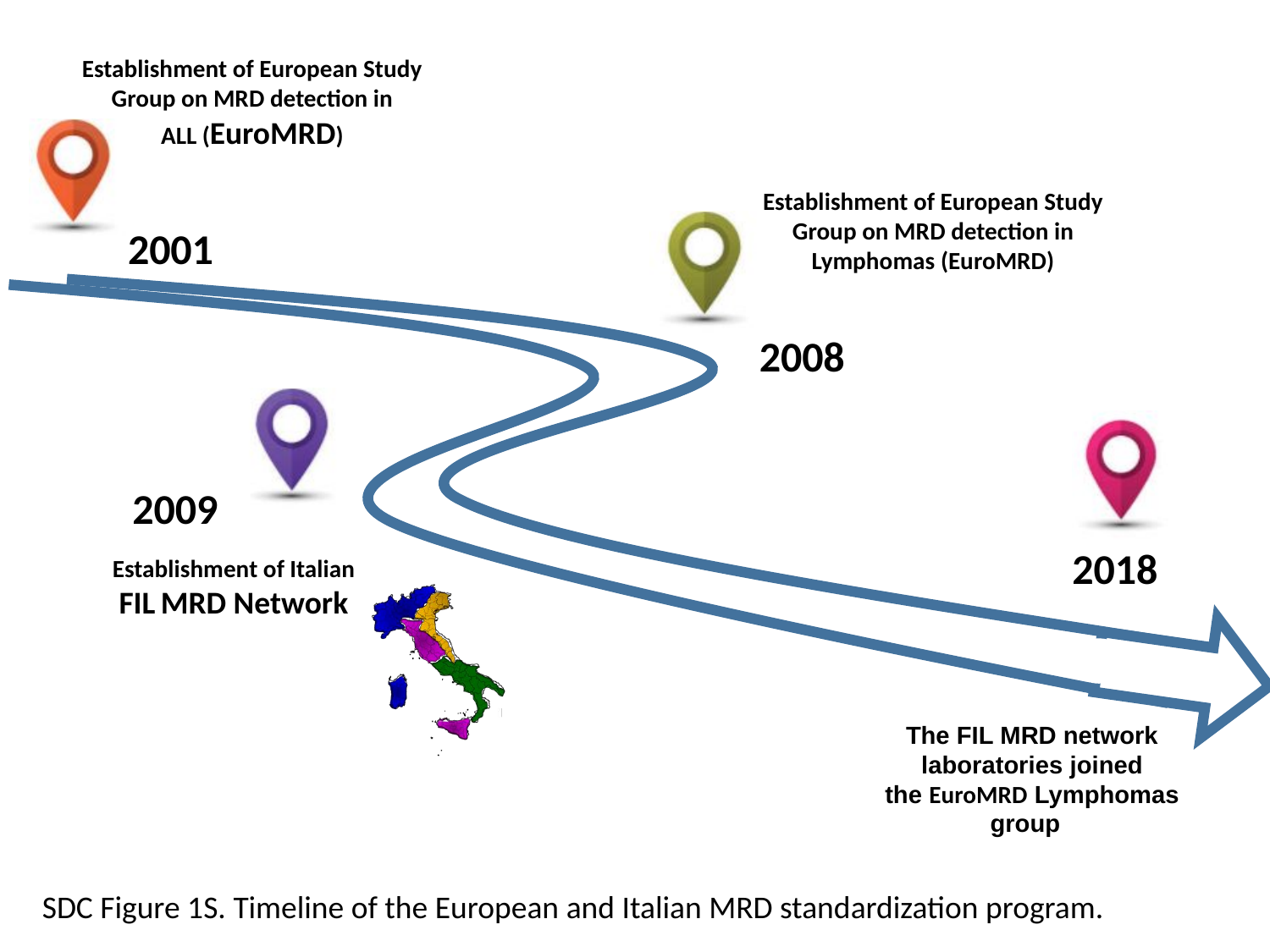

Establishment of European Study Group on MRD detection in ALL (EuroMRD)
Establishment of European Study Group on MRD detection in Lymphomas (EuroMRD)
2001
2008
2009
2018
Establishment of Italian FIL MRD Network
The FIL MRD network laboratories joined the EuroMRD Lymphomas group
SDC Figure 1S. Timeline of the European and Italian MRD standardization program.

## Slide 2
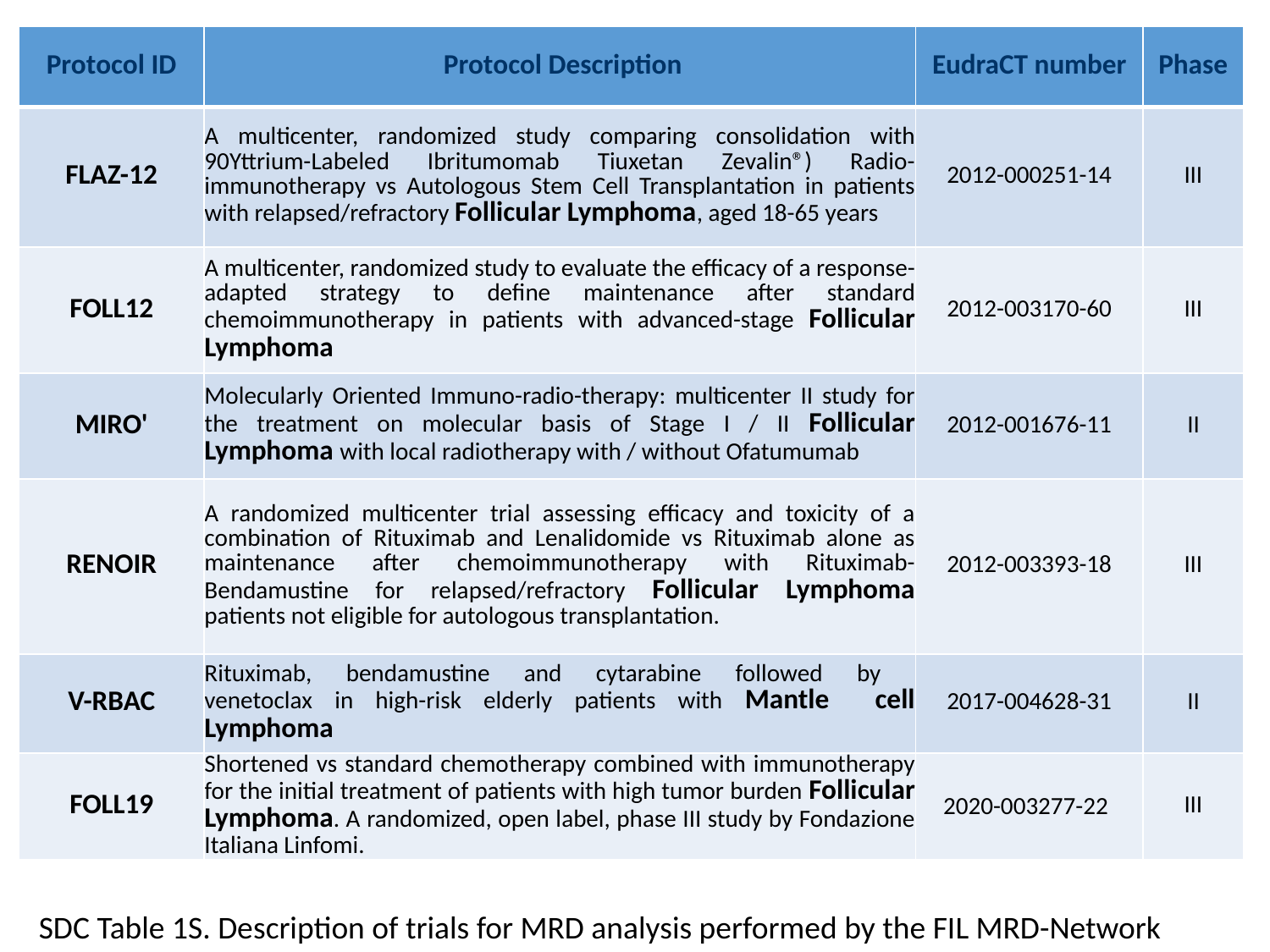

| Protocol ID | Protocol Description | EudraCT number | Phase |
| --- | --- | --- | --- |
| FLAZ-12 | A multicenter, randomized study comparing consolidation with 90Yttrium-Labeled Ibritumomab Tiuxetan Zevalin®) Radio-immunotherapy vs Autologous Stem Cell Transplantation in patients with relapsed/refractory Follicular Lymphoma, aged 18-65 years | 2012-000251-14 | III |
| FOLL12 | A multicenter, randomized study to evaluate the efficacy of a response-adapted strategy to define maintenance after standard chemoimmunotherapy in patients with advanced-stage Follicular Lymphoma | 2012-003170-60 | III |
| MIRO' | Molecularly Oriented Immuno-radio-therapy: multicenter II study for the treatment on molecular basis of Stage I / II Follicular Lymphoma with local radiotherapy with / without Ofatumumab | 2012-001676-11 | II |
| RENOIR | A randomized multicenter trial assessing efficacy and toxicity of a combination of Rituximab and Lenalidomide vs Rituximab alone as maintenance after chemoimmunotherapy with Rituximab-Bendamustine for relapsed/refractory Follicular Lymphoma patients not eligible for autologous transplantation. | 2012-003393-18 | III |
| V-RBAC | Rituximab, bendamustine and cytarabine followed by venetoclax in high-risk elderly patients with Mantle cell Lymphoma | 2017-004628-31 | II |
| FOLL19 | Shortened vs standard chemotherapy combined with immunotherapy for the initial treatment of patients with high tumor burden Follicular Lymphoma. A randomized, open label, phase III study by Fondazione Italiana Linfomi. | 2020-003277-22 | III |
SDC Table 1S. Description of trials for MRD analysis performed by the FIL MRD-Network

## Slide 3
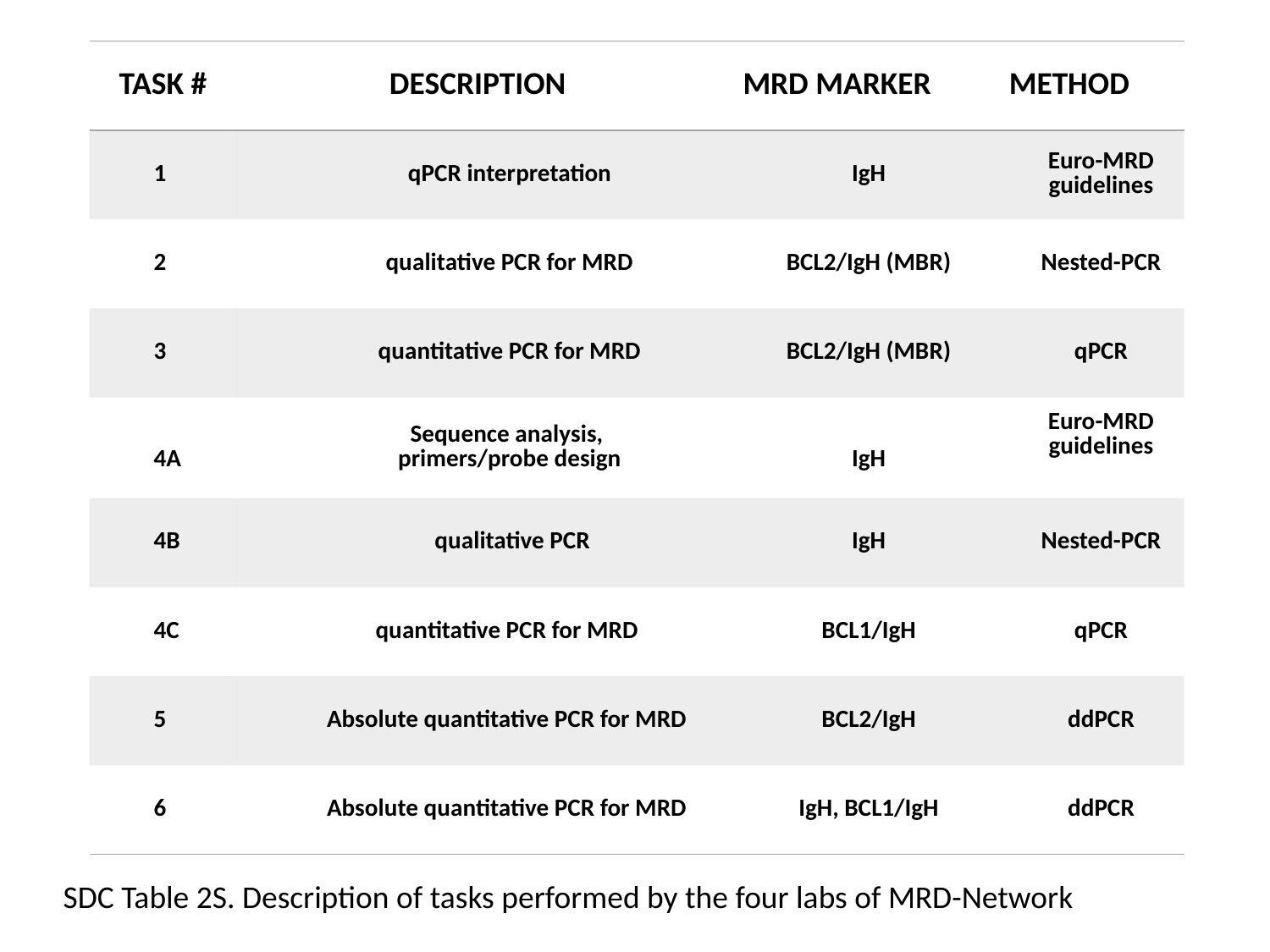

| TASK # | DESCRIPTION | MRD MARKER | METHOD |
| --- | --- | --- | --- |
| 1 | qPCR interpretation | IgH | Euro-MRD guidelines |
| 2 | qualitative PCR for MRD | BCL2/IgH (MBR) | Nested-PCR |
| 3 | quantitative PCR for MRD | BCL2/IgH (MBR) | qPCR |
| 4A | Sequence analysis, primers/probe design | IgH | Euro-MRD guidelines |
| 4B | qualitative PCR | IgH | Nested-PCR |
| 4C | quantitative PCR for MRD | BCL1/IgH | qPCR |
| 5 | Absolute quantitative PCR for MRD | BCL2/IgH | ddPCR |
| 6 | Absolute quantitative PCR for MRD | IgH, BCL1/IgH | ddPCR |
SDC Table 2S. Description of tasks performed by the four labs of MRD-Network

## Slide 4
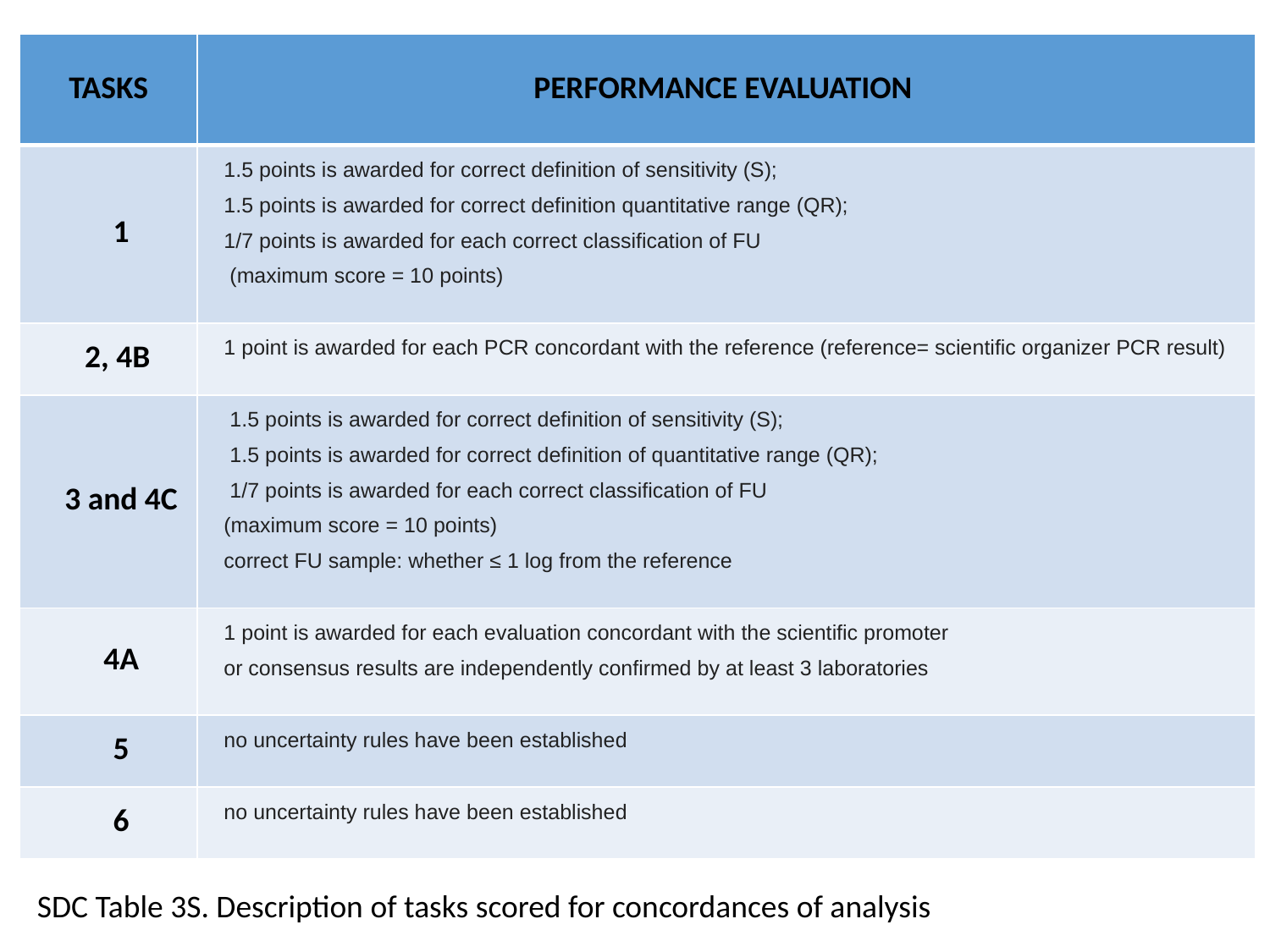

| TASKS | PERFORMANCE EVALUATION |
| --- | --- |
| 1 | 1.5 points is awarded for correct definition of sensitivity (S); 1.5 points is awarded for correct definition quantitative range (QR); 1/7 points is awarded for each correct classification of FU (maximum score = 10 points) |
| 2, 4B | 1 point is awarded for each PCR concordant with the reference (reference= scientific organizer PCR result) |
| 3 and 4C | 1.5 points is awarded for correct definition of sensitivity (S); 1.5 points is awarded for correct definition of quantitative range (QR); 1/7 points is awarded for each correct classification of FU (maximum score = 10 points) correct FU sample: whether ≤ 1 log from the reference |
| 4A | 1 point is awarded for each evaluation concordant with the scientific promoter or consensus results are independently confirmed by at least 3 laboratories |
| 5 | no uncertainty rules have been established |
| 6 | no uncertainty rules have been established |
SDC Table 3S. Description of tasks scored for concordances of analysis

## Slide 5
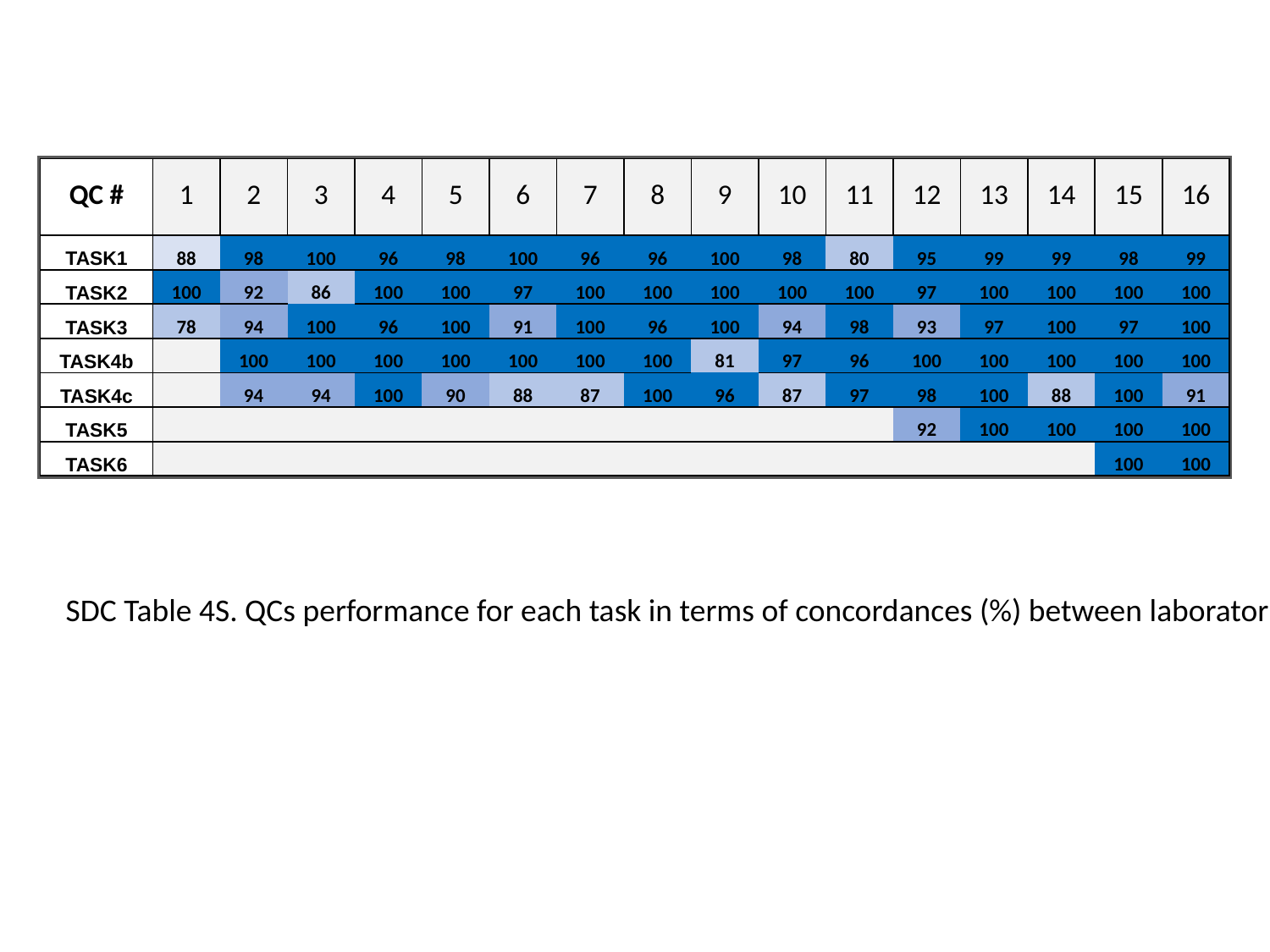

| QC # | 1 | 2 | 3 | 4 | 5 | 6 | 7 | 8 | 9 | 10 | 11 | 12 | 13 | 14 | 15 | 16 |
| --- | --- | --- | --- | --- | --- | --- | --- | --- | --- | --- | --- | --- | --- | --- | --- | --- |
| TASK1 | 88 | 98 | 100 | 96 | 98 | 100 | 96 | 96 | 100 | 98 | 80 | 95 | 99 | 99 | 98 | 99 |
| TASK2 | 100 | 92 | 86 | 100 | 100 | 97 | 100 | 100 | 100 | 100 | 100 | 97 | 100 | 100 | 100 | 100 |
| TASK3 | 78 | 94 | 100 | 96 | 100 | 91 | 100 | 96 | 100 | 94 | 98 | 93 | 97 | 100 | 97 | 100 |
| TASK4b | | 100 | 100 | 100 | 100 | 100 | 100 | 100 | 81 | 97 | 96 | 100 | 100 | 100 | 100 | 100 |
| TASK4c | | 94 | 94 | 100 | 90 | 88 | 87 | 100 | 96 | 87 | 97 | 98 | 100 | 88 | 100 | 91 |
| TASK5 | | | | | | | | | | | | 92 | 100 | 100 | 100 | 100 |
| TASK6 | | | | | | | | | | | | | | | 100 | 100 |
SDC Table 4S. QCs performance for each task in terms of concordances (%) between laboratories

## Slide 6
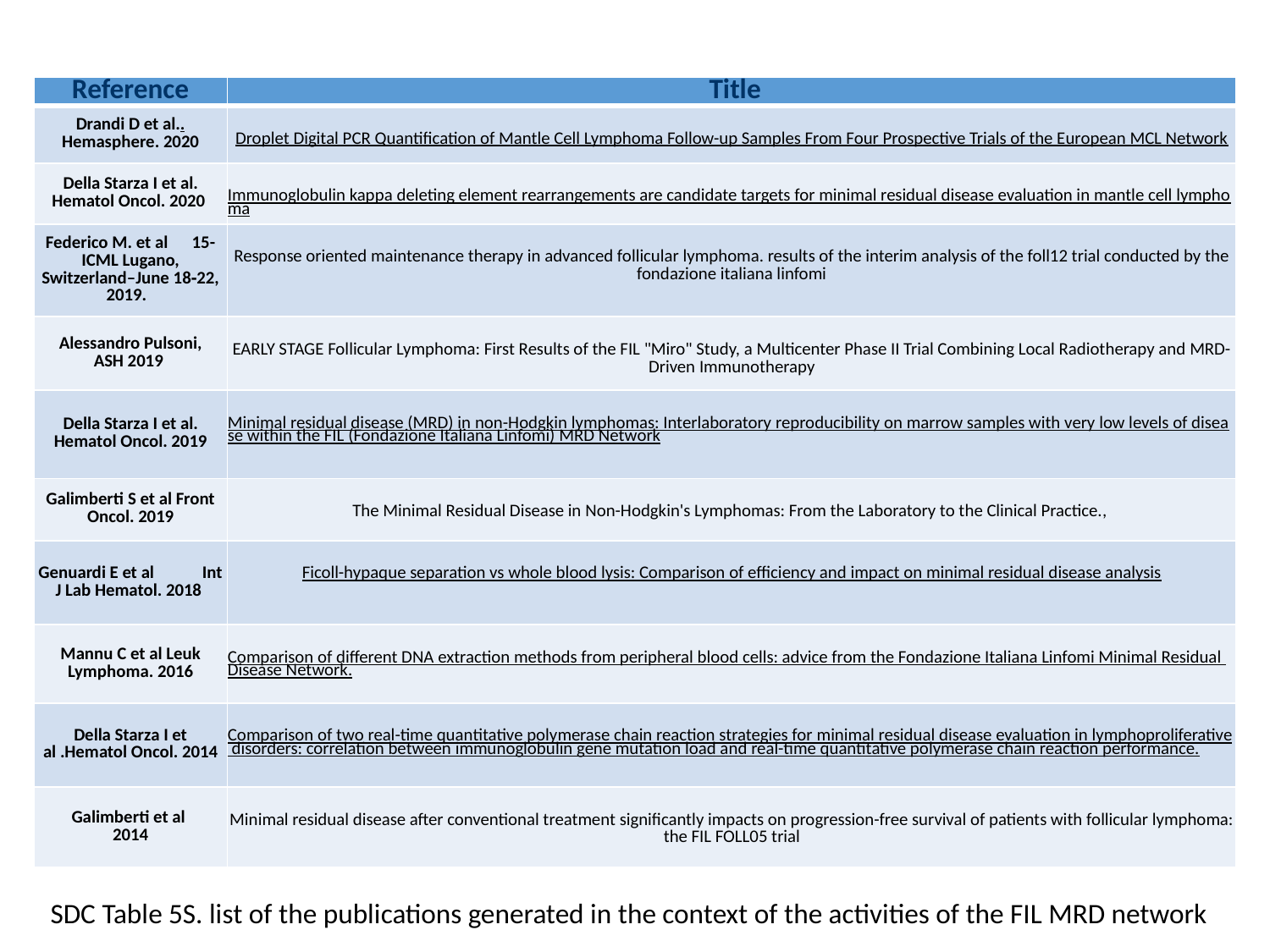

| Reference | Title |
| --- | --- |
| Drandi D et al.. Hemasphere. 2020 | Droplet Digital PCR Quantification of Mantle Cell Lymphoma Follow-up Samples From Four Prospective Trials of the European MCL Network |
| Della Starza I et al. Hematol Oncol. 2020 | Immunoglobulin kappa deleting element rearrangements are candidate targets for minimal residual disease evaluation in mantle cell lymphoma |
| Federico M. et al 15-ICML Lugano, Switzerland–June 18‐22, 2019. | Response oriented maintenance therapy in advanced follicular lymphoma. results of the interim analysis of the foll12 trial conducted by the fondazione italiana linfomi |
| Alessandro Pulsoni, ASH 2019 | EARLY STAGE Follicular Lymphoma: First Results of the FIL "Miro" Study, a Multicenter Phase II Trial Combining Local Radiotherapy and MRD-Driven Immunotherapy |
| Della Starza I et al. Hematol Oncol. 2019 | Minimal residual disease (MRD) in non-Hodgkin lymphomas: Interlaboratory reproducibility on marrow samples with very low levels of disease within the FIL (Fondazione Italiana Linfomi) MRD Network |
| Galimberti S et al Front Oncol. 2019 | The Minimal Residual Disease in Non-Hodgkin's Lymphomas: From the Laboratory to the Clinical Practice., |
| Genuardi E et al Int J Lab Hematol. 2018 | Ficoll-hypaque separation vs whole blood lysis: Comparison of efficiency and impact on minimal residual disease analysis |
| Mannu C et al Leuk Lymphoma. 2016 | Comparison of different DNA extraction methods from peripheral blood cells: advice from the Fondazione Italiana Linfomi Minimal Residual Disease Network. |
| Della Starza I et al .Hematol Oncol. 2014 | Comparison of two real-time quantitative polymerase chain reaction strategies for minimal residual disease evaluation in lymphoproliferative disorders: correlation between immunoglobulin gene mutation load and real-time quantitative polymerase chain reaction performance. |
| Galimberti et al 2014 | Minimal residual disease after conventional treatment significantly impacts on progression-free survival of patients with follicular lymphoma: the FIL FOLL05 trial |
SDC Table 5S. list of the publications generated in the context of the activities of the FIL MRD network
